# siRNA inhibition and not chemical inhibition of Suv39h1/2 enhances pre-implantation embryonic development of bovine somatic cell nuclear transfer embryos

**DOI:** 10.1371/journal.pone.0233880

**Published:** 2020-06-04

**Authors:** Farnoosh Jafarpour, Faezeh Ghazvini Zadegan, Somayyeh Ostadhosseini, Mehdi Hajian, Abbas Kiani-Esfahani, M. H. Nasr-Esfahani

**Affiliations:** 1 Department of Reproductive Biotechnology, Reproductive Biomedicine Research Center, Royan Institute for Biotechnology, ACECR, Isfahan, Iran; 2 Department of Cellular Biotechnology, Cell Science Research Center, Royan Institute for Biotechnology, ACECR, Isfahan, Iran; University of Florida, UNITED STATES

## Abstract

The efficiency of somatic cell nuclear transfer (SCNT) is low due to the strong resistance of somatic donor cells to epigenetic reprogramming. Many epigenetic drugs targeting DNA methylation and histone acetylation have been used in attempts to improve the *in vitro* and *in vivo* development of SCNT embryos. H3K9me3 has been shown to be an important reprogramming barrier for generating induced pluripotent stem cells (iPSCs) and SCNT embryos in mice and humans. In this study, we examined the effects of selective siRNA and chemical inhibition of H3K9me3 in somatic donor cells on the *in vitro* development of bovine SCNT embryos. Chaetocin, an inhibitor of SUV39H1/H2, was supplemented during the culture of donor cells. In addition, the siRNA knockdown of *SUV39H1/H2* was performed in the donor cells. The effects of chaetocin and siSUV39H1/H2 on H3K9me3 and H3K9ac were quantified using flow cytometry. Furthermore, we assessed chaetocin treatment and SUV39H1/H2 knockdown on the blastocyst formation rate. Both chaetocin and siSUV39H1/H2 significantly reduced and elevated the relative intensity level of H3K9me3 and H3K9ac in treated fibroblast cells, respectively. siSUV39H1/H2 transfection, but not chaetocin treatment, improved the *in vitro* development of SCNT embryos. Moreover, siSUV39H1/H2 altered the expression profile of the selected genes in the derived blastocysts, similar to those derived from *in vitro* fertilization (IVF). In conclusion, our results demonstrated H3K9me3 as an epigenetic barrier in the reprogramming process mediated by SCNT in bovine species, a finding which supports the role of H3K9me3 as a reprogramming barrier in mammalian species. Our findings provide a promising approach for improving the efficiency of mammalian cloning for agricultural and biomedical purposes.

## Introduction

Extensive chromatin remodeling plays an indispensable role in different developmental processes, especially after fertilization and during somatic cell nuclear transfer (SCNT) [1–3]. The outcomes of *in vitro* fertilization (IVF) and SCNT are dependent on adequate chromatin remodeling [3].

Despite the marked potential of the SCNT technique for reprogramming terminally differentiated somatic cells into a totipotent state, many studies have shown that this is not very efficient during SCNT procedure [4]. Therefore, the efficiency of SCNT has been found to be low in the majority of mammalian species [5, 6]. Nuclear reprogramming in SCNT-derived embryos is highly error-prone and leads to inadequate early and late embryonic development [7–9]. While the mechanisms underlying incomplete reprogramming remain poorly understood, the epigenetic status of the donor cell is an important biological factor for determining the efficiency of SCNT [10, 11].

Currently, the most resourceful approach involves improving the efficiency of transcriptional reprogramming during SCNT by modifying the epigenetic status of the donor cells and/or reconstructed oocytes using various epigenetic modifiers, such as DNA methyltransferase inhibitors (DNMTis) and histone deacetylase inhibitors (HDACis) [12, 13]. These two categories of epigenetic modifiers induce DNA hypomethylation and histone hyperacetylation, respectively, which lead to the relaxation and accessibility of chromatin template, which facilitates the incorporation of reprogramming factors into the newly introduced chromatin [14–16].

Various DNMTis and HDACis have been extensively used to improve the epigenetic reprogramming in SCNT-derived embryos in different species. Several studies have shown that this approach can significantly increase the efficiency of early and/or full-term development in different species [17–22].

Another approach to improve reprogramming involves targeting histone methylation on lysine residues. However, this approach has received less attention during nuclear reprogramming in SCNT or induced pluripotent stem cells (iPSCs).

In contrast to histone acetylation, histone methylation does not change the charge of lysine sites in histones; more importantly, histone methyltransferase enzymes (HMTs) are highly specific and only target certain residues on histones [23]. Biochemical studies have revealed that histone lysine methylation is associated with either transcriptional activation or repression, depending on the lysine residue that is modified [24].

One of the most well-known sites of histone methylation is lysine 9 on histone H3 (H3K9). Histone methyltransferase enzymes SUV39H1, SUV39H2, and SETDB1 carry out the tri-methylation of H3K9me3, which is associated with heterochromatin and gene silencing [25].

Zhang et al. demonstrated that reprogramming-resistant regions (RRRs) in SCNT embryos are enriched for H3K9me3 in donor cells and its removal by ectopically expressed Kdm4d or siRNA inhibition of SUV39H1/H2 markedly improves SCNT efficiency [26]. Thus, H3K9me3 has been identified as an epigenetic barrier during nuclear reprogramming for generating SCNT embryos and iPSCs in both mice and humans, wherein the removal of this epigenetic barrier markedly improved the efficiency of SCNT and iPSCs [26–28].

The removal of H3K9me3 through *KDM4* overexpression has also been investigated in bovine species. *KDM4E* has been shown to function as a crucial epigenetic regulator during embryonic genome activation (EGA) and is responsible for mediating epigenetic barriers during SCNT reprogramming [29]. In addition, *KDM4B*-bovine fibroblasts significantly reprogrammed better into cloned blastocysts than control donor cells. However, removing H3K9me3 during embryo culture did not increase post-implantation development [30].

Given the high specificity of HMT enzymes, it is more difficult to define chemical inhibitors that would selectively inhibit HMTs. Chaetocin is a fungal histone lysine methyltransferase inhibitor that specifically inhibits the histone methyltransferase SUV39H1/H2 [31].

In this study, we aimed to elucidate the role of H3K9me3 during bovine SCNT, through the removal of this epigenetic mark by either using chaetocin or downregulating the *SUV39H1/H2* in the fibroblast somatic donor cells using siRNA.

## Materials and methods

### Media and reagents

All reagents and media were obtained from Sigma Chemical Co. (St. Louis, MO) and Gibco (Grand Island, NY, USA), respectively, unless specified otherwise.

All animal experiments were approved by the Institutional Review Board and Institutional Ethical Committee of the Royan Institute. The bovine ovaries used in the study were obtained from cows at a local slaughterhouse (Fasaran, Isfahan), with the permission of the manager of the slaughterhouse and the agreement of veterinary organization.

### Somatic donor cell preparation

#### Fibroblast cell collection and culture

Bovine fetal fibroblast cells (BFFs) were isolated from a 2-month-old female embryo. Briefly, after removing the head, limbs, and viscera, the other remaining tissues were washed in phosphate buffered saline without calcium and magnesium (PBS). The tissue was finely minced using a sterile razor blade until it became possible to pipette. Subsequently, the tissue was dissociated with 0.25% trypsin/EDTA (Gibco, Invitrogen). After the inactivation of trypsin, the cell suspension was washed, centrifuged, and cultured in Dulbecco’s modified Eagle medium F-12 (DMEM/F-12) supplemented with 10% fetal bovine serum (FBS) and 1% penicillin-streptomycin at 37.5°C and 5% CO_2_ in a humidified atmosphere. After reaching confluency, the BFFs were harvested and frozen in liquid nitrogen. Frozen cells were thawed and used for various assessments.

#### Preparation of chaetocin and trichostatin A (TSA) solution

Commercially available chaetocin (SUV39H1/H2 histone methyltransferase inhibitor; Sigma, C9492) and TSA (histone deacetylase inhibitor; Sigma, T8552) were dissolved in DMSO, formulated to 0.5 and 0.2 mM stock solutions, respectively, and stored at −20°C. DMEM/F-12 was used to dilute the stock solutions to obtain the desired working solution.

#### Treatment of donor cells with chaetocin and/or TSA

At passage three, the fibroblast cells were treated with chaetocin ranging from 10 to 375 nM for 3 and 5 days, or 1 μM TSA for 1 day, based on our previous study [18]. To determine the synergistic effect of chaetocin and TSA, the cells were treated with chaetocin for 48 h; subsequently, the cells were treated with a combination of chaetocin and TSA for a further 24 h. At the end of each treatment, the treated cells, along with their corresponding untreated groups, were used for the analysis of the different cellular characteristics and SCNT, as described below. All experiments were performed in triplicate. Since chaetocin is relatively unstable, the culture media were refreshed every 24 h.

### Cytotoxicity assessment

The toxicity of various concentrations of chaetocin, TSA, and a combination of chaetocin and TSA in the fibroblast cells were determined using the 3-(4, 5-dimethylthiazol-2-yl)-5-(3-carboxymethoxyphenyl)-2-(4-sulfophenyl)-2H-tetrazolium (MTS) assay. Briefly, 5000 BFFs were cultured in DMEM/F-12 supplemented 10% FBS in 96-well dishes. After 24 h, DMEM/F-12 + 10% FBS supplemented with varying concentrations of the desired drug was added to the cultured cells and incubated for 1, 2, and 3 days. Next, MTS was added to each well and incubated for 4 h at 37°C. The absorbance ratio of the treated cells relative to the control was measured at 492 nm using a multi-well spectrophotometer. All analyses were measured in three independent replications, where each replication consisted of triplicate samples.

### siRNA design

The mRNA sequence of the bovine target genes (*SUV39H1* and *SUV39H2*) was obtained from the NCBI gene bank (GenBank) (accession no. NM_001046264.2 and NM_001037479.2). The siRNAs were designed using the online tool BLOCK-iT, WIZARD and Dharmacon RNAi designer to select specific target sequences for siRNAs. Initially, all the siRNAs were examined by BLAST analysis to prevent the inclusion of any potential non-specific targets. A scrambled (SCR) siRNA that would not target other bovine genes was also synthesized to exclude off-target effects. The primer sequences of *SUV39H1* and *SUV39H2* siRNA are shown in S1 Table.

### Knockdown of *SUV39H1* and *SUV39H2* in BFFs by siRNA transfection

The siRNA transfection of BFFs was performed according to the methods reported by Matoba et al. [26]. To this end, the siRNAs against bovine *SUV39H1 and SUV39H2* (S1 Table) were diluted in nuclease-free water at 50 μM stock solutions. siRNAs were introduced into the BFFs with Lipofectamine 3000 (#3000–001; Life Technologies). Briefly, 1 × 10^5^ BFFs were seeded into a 24-well plate (day 0). After 24 h, the BFFs were transfected with 5 pM siRNAs Lipofectamine 3000 (day 1). After 24 h, the culture media were refreshed (day 2). On day 3, the BFFs were passaged into 24-well plates at the density of 1 × 10^5^ cells. Transfection was repeated once, as described above (day 4). Next, 48 h after the second transfection (day 6), the transfected BFFs were used for further assessments (flow cytometry, RT-qPCR, or SCNT). In addition, to determine the synergistic effect of siSUV39H1/H2 and TSA in SCNT, 24 h after the second transfection (day 5), the transfected BFFs were treated with TSA for 24 h and then used for SCNT.

In a separate experiment, the relative intensity of H3K9me3 was evaluated in both single and double transfected cells using flow cytometry.

### Semi-quantitative assessment of epigenetic marks in fibroblast cells: Chemically-treated and siRNA-transfected cells

The respective effects of non-toxic doses of chaetocin on two epigenetic marks, namely H3k9me3 and H3K9ac in BFFs and H3K9me3 (Single and double transfected cells) and H3K9ac in *SUV39H1/H2* siRNA-transfected fibroblast cells, were assessed using flow cytometry by measuring the fluorescence intensity of the complexes between DNA/histones with primary and secondary antibodies in the cells, as described previously [18]. Briefly, after either treating the BFFs with non-toxic concentrations of chaetocin or the siRNA transfection of BFFs, the cells were fixed with 4% paraformaldehyde (PF). Permeabilization was carried out using 1% Triton X-100 in PBS for 30 min at room temperature (RT). To block non-specific binding sites, the cells were incubated in a blocking solution (PBS + 3% bovine serum albumin) for 2 h at RT. Subsequently, the cells were incubated with primary antibodies (S2 Table) overnight at 4°C. The following day, the cells were washed extensively and incubated with the corresponding secondary antibodies (S2 Table) for 45 min at 37°C. After washing, ten thousand cells were collected with FACS-Caliber and analyzed using CELL QUEST 3.1 software (Becton Dickinson, USA).

### Gene expression analysis in chemically-treated and siRNA-transfected BFFs

For the RNA isolation and quantitative real-time PCR (qRT-PCR) analysis of BFFs treated with chaetocin (20 nM) for 3 days, the siRNA-transfected BFFs, and the control BFFs, the total RNA was extracted using an RNeasy Mini Kit (Qiagen). The samples were then treated with DNaseI (Fermentas) to remove any contaminating genomic DNA. cDNA synthesis was performed by adding 1 μg of total RNA to the random hexamer primer in a RevertAid™ H First Strand cDNA Synthesis Kit (Fermentas). SYBR green (TaKaRa, Japan) was used for real-time PCR analysis in a thermal Cycler Rotor-Gene 6000 (Corbett, Australia). For each reaction, the PCR mixture was comprised of 5 μl of Rotor-Gene SYBR Green PCR Master Mix (TaKaRa), 12.5 ng of cDNA, and 1.5 pmol of each primer at a final volume of 10 μl. The analysis of gene expressions was carried out using the ΔΔCT method. The relative levels of expression were normalized to the expression of the *GAPDH* gene. The sequences of the primers used for the real-time PCR analysis are provided in S3 Table.

### *In vitro* embryo production

#### Preparation of bovine ovaries and oocyte collection

The ovaries were obtained from cows at a local slaughterhouse (Fasaran, Isfahan) with the permission of the slaughterhouse manager and the agreement of veterinary organization. The slaughterhouse ovaries were collected between 2 pm and 4 pm and transported to the laboratory in saline supplemented with 50 IU/ml penicillin and 50 g/ml streptomycin at 15–17°C before 6 pm. Upon arrival, the ovaries were immediately washed, trimmed, and stored at 15°C until the harvesting of the oocytes, based on previously reported protocols [32]. The cumulus-oocyte complexes (COCs) were aspirated from 2–8 mm follicles using an 18 gauge needle attached to a vacuum pump. Only oocytes possessing a homogenous cytoplasm and at least three layers of compact cumulus cells were selected for *in vitro* maturation (IVM).

#### IVM of bovine oocytes

The IVM of oocytes was carried out according to the method described by Jafarpour et al. [33]. Briefly, the selected COCs were washed in HEPES tissue culture medium 199 (H-TCM), followed by washing in TCM medium supplemented with sodium pyruvate, 10% FBS, 10 μg/ml follicle-stimulating hormone (FSH) (F8174; Sigma), 10 μg/ml luteinizing hormone (LH) (L5269; Sigma), 100mM 17β-estradiol (E2) (E4389; Sigma), 0.1 mM cysteamine (M9768; Sigma), 10 ng/ml epidermal growth factor (EGF) (E4127; Sigma), and 100 ng/ml insulin-like growth factor 1 (IGF1) (291-G1; R&D). The COCs were then transferred and cultured in groups of 10 into 50 μl droplets of maturation medium (MM) and incubated for 22 h at 38.5°C in a humidified 5% CO_2_ atmosphere under mineral oil.

#### In vitro fertilization (IVF) of bovine

In this study, IVF was carried out as the control. The IVF procedure was carried out as described previously [34]. Frozen semen straws were thawed (30 s in air and then 1 min in a 37°C water bath). Subsequently, the semen was centrifuged at 300 *g* for 5 min at RT to remove the cryoprotectant. The semen pellet was then layered on a discontinuous PureSperm^®^ gradient (40% over 80% prepared in H-TCM + 10% FBS), and the motile sperms were collected after centrifugation at 100 *g* for 15 min at RT. The prepared samples were washed in HEPES buffered Tyrode’s albumin lactate pyruvate (TALP) medium and 1 × 10^6^/ml sperms were co-incubated with 10 COCs in 50 μl of IVF-TALP (TALP supplemented with 6 mg/ml BSA and 20 μg/ml heparin) for 18 h at 38.5°C in a humidified atmosphere with 5% CO_2_. After 18 h, the presumptive zygotes were denuded of cumulus cells and cultured in modified synthetic oviduct fluid without glucose and serum (mSOF^-^) for three days. After the third day, the cleaved embryos were transferred to mSOF in the presence of charcoal-stripped serum (5%) and glucose (1.5 mM) (mSOF^+^) for 4–5 days at 38.5°C in a humidified air with 5% CO_2_ and 5% O_2_ under mineral oil. The resulting blastocysts were selected for subsequent gene expression analysis.

#### Embryo production by SCNT in bovine

Matured oocytes were denuded by vortexing inside H-TCM supplemented with 300 IU/ml hyaluronidase for 3 min. The denuded oocytes were exposed to 5 mg/ml pronase for several seconds, followed by deactivation with H-TCM + 20% FBS for 20 min to remove the zona pellucida. Oocyte enucleation was performed through manual oocyte enucleation using a fine pulled Pasteur pipette [35]. Briefly, the zona-free oocytes were incubated in TCM supplemented with 4 μg/ml demecolcine for 1 h at 38.5°C. Next, the cytoplasmic protrusion, containing metaphase II (MII) spindle, was removed using a hand-held manual oocyte enucleation pipette. For nuclear replacement, the enucleated oocytes were transferred to dishes containing droplets of H-TCM, supplemented with 10 mg/ml phytohemagglutinin, and well-rounded fibroblast cells were attached to the membrane of the enucleated oocytes. Next, the couplets in the fusion buffer, free of Ca^2+^ and Mg^2+^ (290 mOsm), were electrofused using a sinusoidal electric current (7 V/cm) for 10 s, followed by two direct currents (1.75 kV/cm for 30 μs and 1 s delay). After 30 min, oocyte activation was induced by the incubation of reconstructed oocytes with 5μM Ca-ionophore for 5 min, followed by incubation for 4 h with 2 mM 6-dimethylaminopurine (6-DAMP). The activated reconstructed oocytes were then cultured inside the wells, which contained mSOF^+^ at 38.5°C in a humidified atmosphere with 5% CO_2_ and 5% O_2_ for 7 days under mineral oil.

To preclude the aggregation of zona-free embryos, 20 μl droplets of mSOF^+^ were prepared under mineral oil in 3-cm Grainer^®^ dishes. Six small wells were created by gently pressing a sterile steel rod with a round tip (500-μm diameter) to the bottom of the culture dish. The activated reconstructed oocytes were then placed in separate wells.

### RNA extraction and reverse transcription in embryos

This technique was carried out as previously described [33]. Briefly, pools of bovine blastocysts (n = 6) in various treatment groups were used for RNA extraction using a RNeasy Micro Kit (catalogue no. 74004; Qiagen). Reverse transcription was immediately performed using a QuantiTect Reverse Transcription Kit (catalogue no. 205311; Qiagen). The resulting cDNA was aliquoted and stored at −80°C.

The PCR reactions were prepared using SYBR Green Master (catalogue no. 04673514001; Roche). Each reaction consisted of 1.5 μl of cDNA, 7.5 μl of 2× SYBR Green PCR master mix, 0.9 μl of each primer pair, and 5.1 μl of ultrapure water. The reactions were performed in triplicate and repeated three times. The housekeeping gene *β-actin* was used as the reference gene. The amplification conditions used were as follows: pre-denaturation at 95°C for 10 min, followed by 45 amplification cycles of 95°C denaturation for 15 s, 60°C annealing for 10 s, and 72°C extension for 20 s. Three technical replicates were performed in each of the PCR reactions, which were repeated a total of three times. The ΔΔC_T_ method was used to estimate the fold changes between the genes of interest (2^ΔΔCT^) following RT-qPCR. The value comparative threshold cycle (CT) denotes the threshold cycle. ΔC_T_ was calculated as follows: C_T_ (of the target gene) − C_T_ (of the reference gene). The fold change in the gene expression was calculated using the 2^-ΔΔCT^ method, where ΔΔC_T_ was calculated as ΔC_T_. The sequences of the primer used for real-time PCR analysis are provided in S3 Table.

### Statistical analysis

All experiments were repeated at least in triplicate. When possible, the experimental data were presented as the mean ± standard error of the mean (S.E.M.). SPSS (version 16.0) was used for the one-way analysis of variance (ANOVA) of all data, except for gene expression in transfected cells, which was analyzed using Student’s t-test. P-values <0.05 were considered statistically significant. To explore differences between multiple groups, ANOVA was followed by Tukey post-hoc test

## Results

### High concentrations of chaetocin inhibit the proliferation of BFF cells

**Fig 1:**
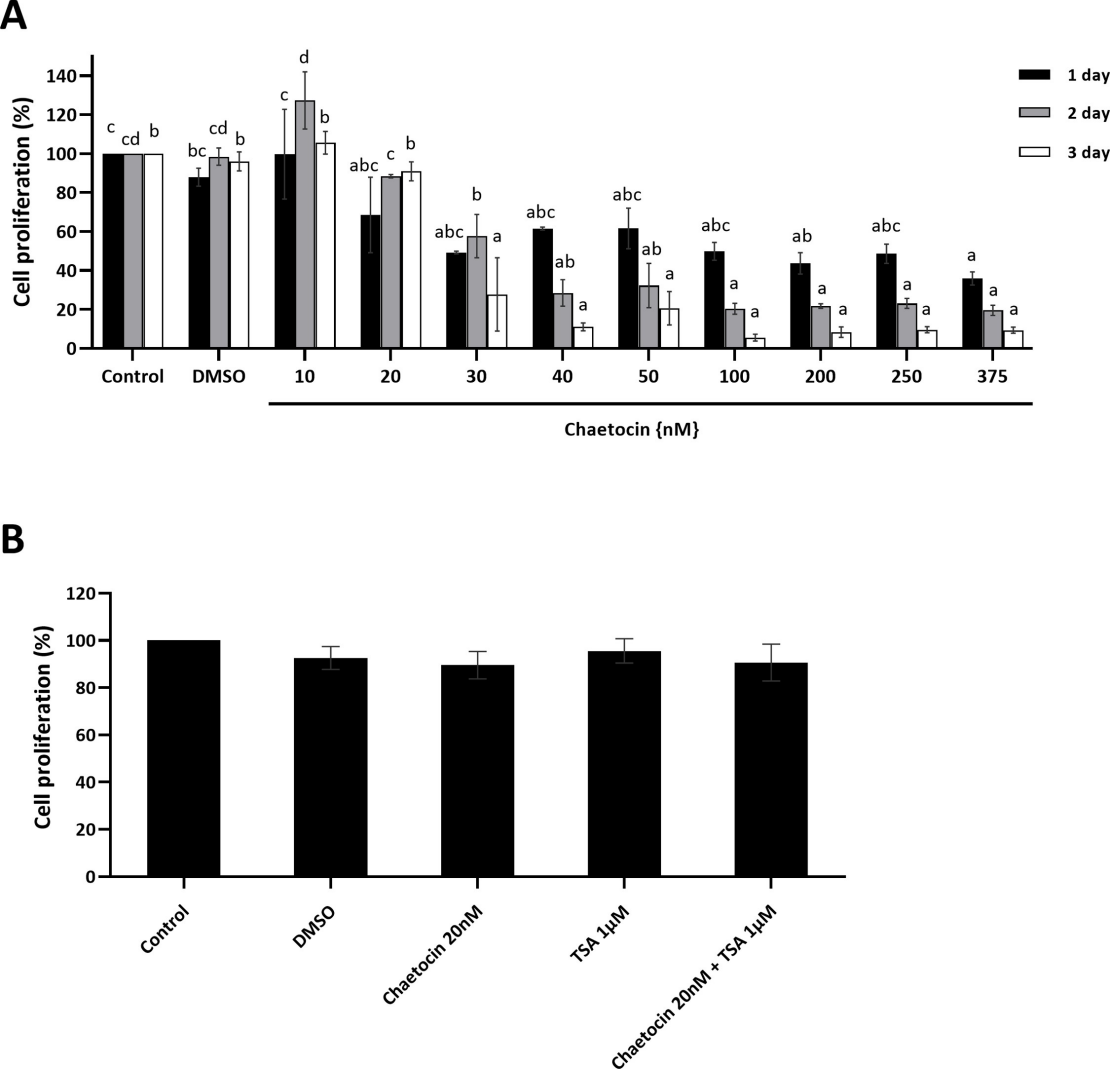
The effects of chaetocin on the proliferation of BFF cells. (A) Cell proliferation of BFF cells exposed to chaetocin–time course and dose-dependence study. BFF cells were exposed to chaetocin at the indicated concentrations for 1 (black bar), 2 (gray bar), and 3 days (white bar). (B) Cell proliferation of BFF cells exposed to chaetocin 20nM, trichostatin A (TSA) 1μM, and combination of chaetocin and TSA for 3 days. The MTS test was performed after indicated times of treatment. The results were normalized to control cells and are presented as the mean ± standard error of mean. DMSO (as a solvent) was used as a control. Different letters within one time point are significantly different at *P*< 0.05.

To determine the effect of chaetocin on the proliferation of BFF cells and the safe concentration range for chaetocin, an MTS assay was performed at 1, 2, and 3 days after drug incubation. None of the chaetocin concentrations affected the proliferation rate after 24 hours (*P* > 0.05), except for 375 nM. Low concentrations (10 and 20 nM) of chaetocin did not reduce cell proliferation after 24 and 36 h (*P* > 0.05), while high concentrations (30–375 nM) resulted in a marked decrease in the cell proliferation rate after 24 and 48 h (*P* < 0.05) (Fig 1A). Based on these findings, we tested the effects of chaetocin (20 nM) in combination with TSA (1 μM) on the proliferation rate of treated BFF cells. Our results did not show any synergistic effect on the inhibition of cell proliferation (Fig 1B), suggesting that chaetocin was cytotoxic in BFF cells only at high concentrations.

### Chaetocin results in global changes to H3K9 trimethylation and H3K9 acetylation in BFF cells

**Fig 2:**
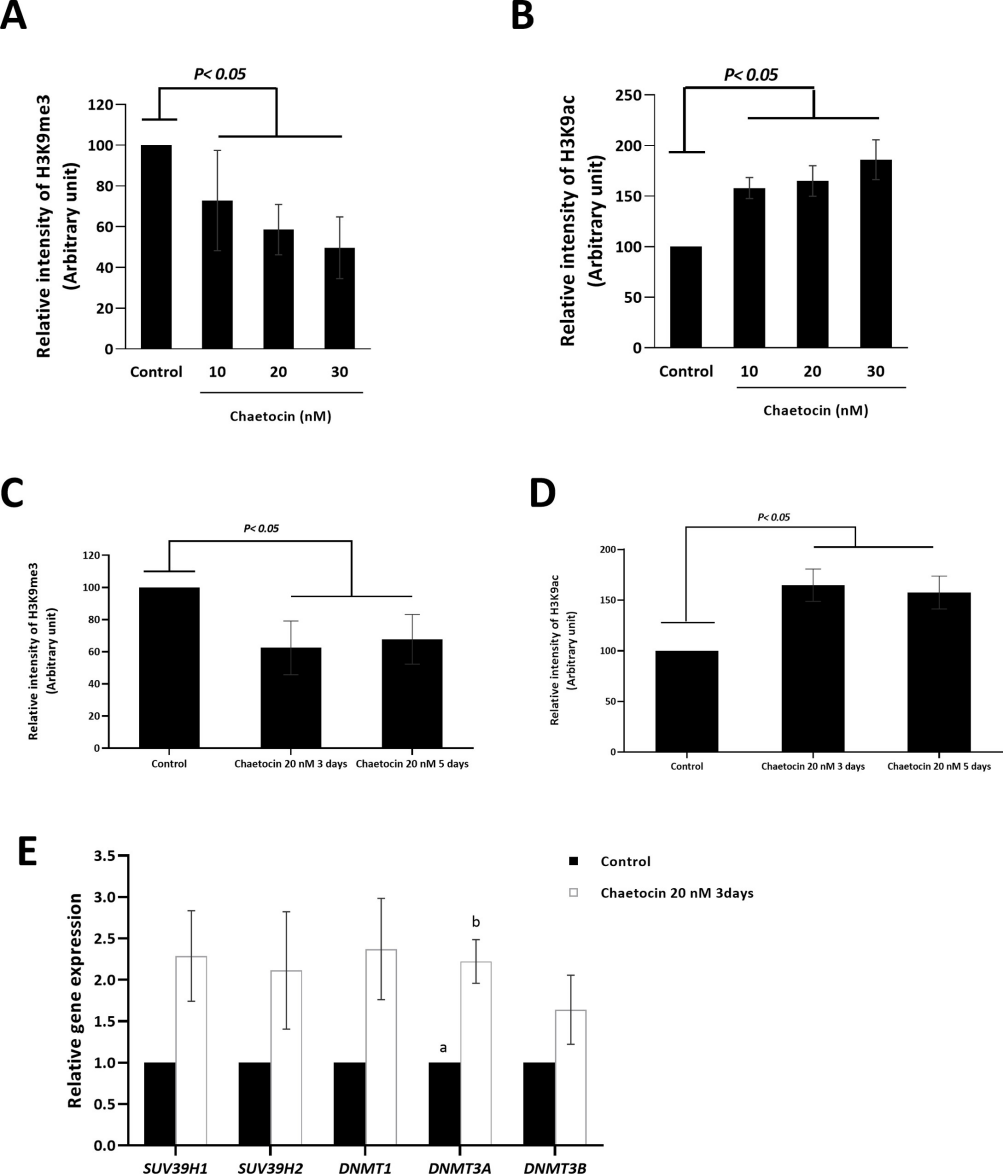
The effects of chaetocin on the histone modifications in the BFF cells Flow cytometry assay for the relative levels of (A) H3K9me3 and (B) H3K9ac was performed on the BFF cells treated with various concentrations of chaetocin for 3 days. Comparison the relative levels of (C) H3K9me3 and (D) H3K9ac using flow cytometry in the BFF cells treated with 20 nM chaetocin for 3 and 5 days. (E) Real-time reverse-transcriptase PCR gene expression analysis of mRNA of *SUV39H1/H2* and *DNMT* family in BFF cells treated with 20 nM chaetocin for 3 days. Fold-change values were calculated from three biological replications following normalization to *GAPDH*. Different letters indicates a significant difference between the groups at *P*< 0.05.

Greiner et al. reported that chaetocin specifically inhibits SUV39H1/2, and reported a reduction in the rate of H3K9 trimethylation in chaetocin-treated cells [31]. The impact of a safe concentration of chaetocin (10 and 20 nM) and a concentration of 30 nM on global H3K9 trimethylation and H3K9 acetylation in BFF cells was investigated using quantitative flow cytometry. Growing BFF cells were treated with chaetocin (10, 20, and 30 nM) for 3 days, resulting in reduced levels of H3K9 trimethylation (Fig 2A) and increased levels of H3K9 acetylation (Fig 2B) (*P* < 0.05). We then extended the time of the 20 nM chaetocin treatment to 5 days and assessed the relative intensity of H3K9me3 and H3K9ac using flow cytometry. Our result revealed that extending the time of chaetocin treatment from 3 days to 5 days did not result in an increase in the effect of treatment on the relative intensity of H3K9me3 (Fig 2C) or H3K9ac (Fig 2D) (*P* > 0.05). These results confirm that chaetocin plays a role in the modification of the epigenetic profile of the treated BFF cells.

Both H3K9 trimethylation and H3K9 acetylation are involved in the regulation of gene expression, namely reduced levels of H3K9 trimethylation and increased levels of H3K9 acetylation, associated with increased gene expression [24]. Therefore, we investigated whether global K3K9 hypomethylation and hyperacetylation levels in BFF cells increased the efficiency of SCNT in bovine species.

### Chaetocin does not affect the expression of DNA methyltransferase family

We assessed the expression of *SUV39H1*, *SUV39H2*, *DNMT1*, *DNMT3A*, and *DNMT3B* in chaetocin-treated (20 nM) BFF cells (3 days) and found a non-significant increase in mRNA expression of all genes except for *DNMT3A*, which demonstrated a significant increase (Fig 2E).

### Chaetocin does not improve the developmental competence of bovine SCNT embryos

Based on the findings reported by Zhang [27, 28], we sought to examine the effects of chaetocin, a small molecule inhibitor of SUV39H1/H2, on the developmental competence of bovine SCNT embryos in terms of cleavage and blastocyst rate. To this end, the BFF cells were treated with 10 and 20 nM chaetocin for 3 days, and the control group was treated with 0 nM chaetocin. As shown in Table 1, no improvement was observed in the cleavage or blastocyst yield after treatment with 10 nM chaetocin (86.12±3.58 and 11.41±2.14) and 20 nM chaetocin (82.87±1.92 and 9.31±1.78) compared to the control group (80.35±2.45 and 9.62±1.25) (*P* > 0.05), respectively. These results may be related to the non-specific nature of chaetocin, a finding that has been previously reported by Cherblanc et al. [36] and needs more investigation.

**Table 1:** The effects of cell donor treatment with chaetocin on the developmental competence of the SCNT derived embryos.

Groups	Treatment		No. of oocytes	Embryo development	
	Chaetocin (nM)	TSA (μM)		% of Cleaved embryos ± SEM	% of blastocysts on day7 ± SEM
SCNT- Control	-	-	951	80.35±2.45 a	9.62±1.25 a
SCNT- Chaetocin 10	10	-	567	86.12±3.58 a	11.41±2.14 a
SCNT- Chaetocin 20	20	-	847	82.87±1.92 a	9.31±1.78 a
SCNT- TSA 1μM	-	1	424	87.05±2.48 a	17.5±0.98 b
SCNT- Chaetocin + TSA	20	1	800	81.86±3.96 a	16.1±1.18 b

Values are presented as mean ± standard error of mean. Different letters indicate significant differences between groups at *P*< 0.05.

Many researchers have demonstrated that the treatment of donor cells and/or reconstructed oocytes with TSA, as an HDAC inhibitor, can improve the developmental competence of SCNT embryos in various species [12, 18]. To determine the synergistic effects of chaetocin and TSA, BFF cells were treated with a combination of chaetocin and TSA. Treatment with TSA alone significantly increased the blastocyst yield (17.5±0.98) of BFFs compared to that of the control group (9.62±1.25) (*P* < 0.05) (Table 1). Combined treatment (chaetocin + TSA) did not result in a further increase of the blastocyst rate (16.1±1.18) in comparison with treatment with TSA alone (17.5±0.98), and were statistically similar to each other (*P*> 0.05) (Table 1). This result indicates that chaetocin and TSA do not have an additive effect on the SCNT efficiency of BFFs during the pre-implantation stage.

### siSUV39H1/H2 transfection in fibroblast cells decreases H3K9 trimethylation, increases H3K9 acetylation, and represses DNA methyltransferase expression

**Fig 3:**
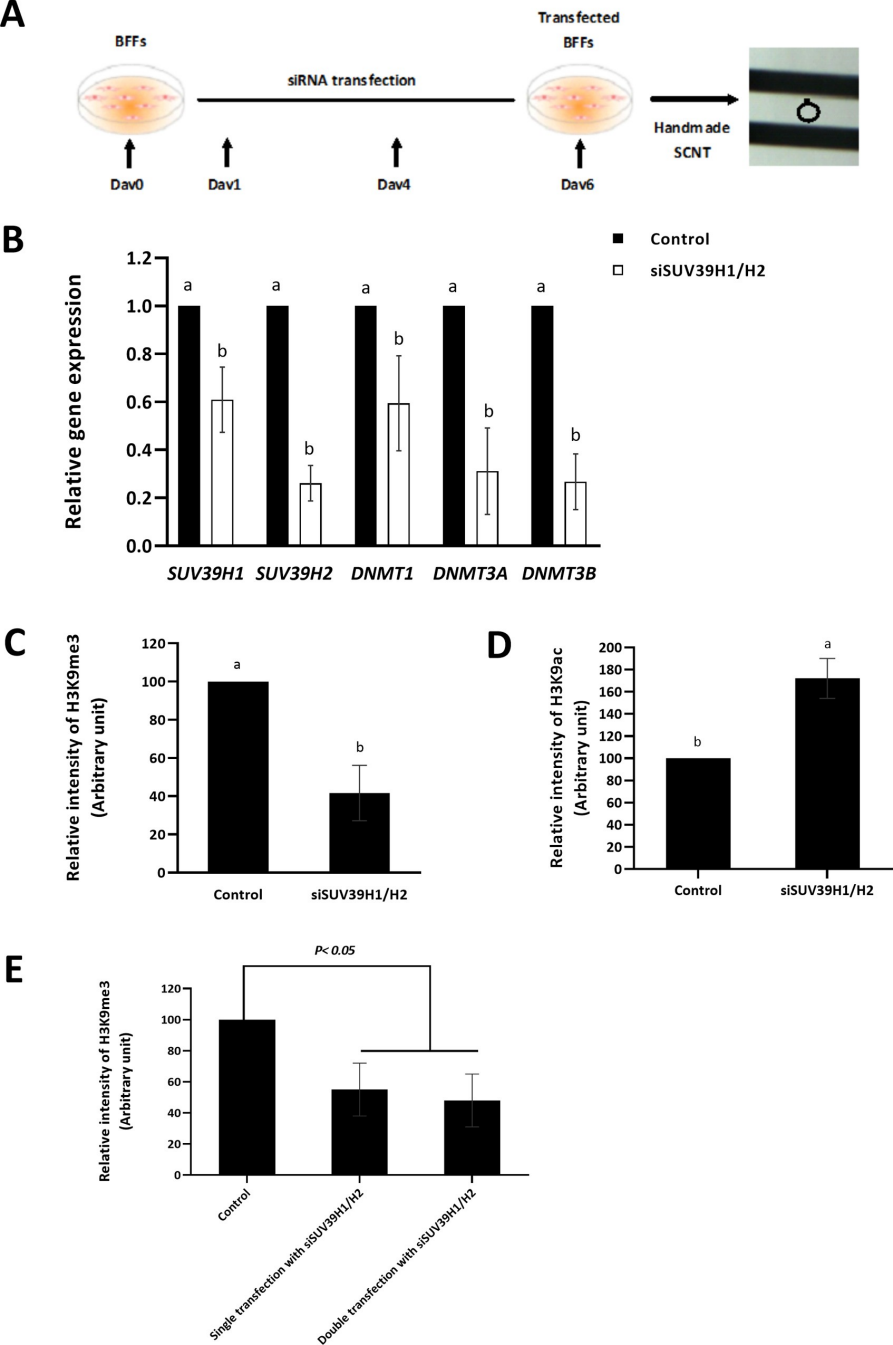
Removal of H3K9me3 by siRNA *SUV39H1/H2* transfection in fibroblast cells. (A) Schematic illustration of siRNA SUV39H1/H2 transfection of the BFF cells used for SCNT. (B) Real-time reverse-transcriptase PCR gene expression analysis of mRNA of *SUV39H1/H2* and *DNMT* family in siSUV39H1/H2 transfected and non-transfected BFF cells. Fold-change values were calculated from three biological replications following normalization to *GAPDH*. The relative levels of (C) H3K9me3 and (D) H3K9ac in siSUV39H1/H2 transfected (double transfection) and non-transfected BFF cells. (E) The relative intensity of H3K9me3 in once and double siRNA transfected cells. Values are presented as mean ± standard error of mean. Different letters indicate significant differences between the groups at *P* < 0.05.

Having demonstrated that the chemical inhibition of SUV39H1/H2 with chaetocin did not improve the SCNT efficiency of BFFs, we investigated whether H3K9 trimethylation could be reduced by *SUV39H1/H2* siRNA inhibition (Fig 3A). Our results indicated that the transfection of fibroblast cells with a mixture of siSUV39H1 and siSUV39H2 reduced the expression of *SUV39H1* and *SUV39H2* to 60.99% and 26.14%, respectively (*P* < 0.05) (Fig 3B). Previous studies have shown that there is an association between DNA methyltransferase enzymes and SUV39H1/H2 (Fuks et al., 2003). The subsequent RT-qPCR analysis revealed a significant knockdown of DNA methyltransferase family, including *DNMT1*, *DNMT3A*, and *DNMT3B* (*P* < 0.05), which is in agreement with previously reported findings (Fig 3B).

To further determine the effects of *SUV39H1/H2* knockdown on the corresponding epigenetic marks, i.e. H3K9 trimethylation, we quantified the levels of H3K9me3 in siRNA-transfected cells by flow cytometry. These results demonstrated a 41.69% reduction in the relative intensity of H3K9me3 levels in the siRNA-transfected cells compared with the control cells (*P* < 0.05) (Fig 3C). We then assessed the relative intensity of H3K9ac in the siRNA-treated cells using flow cytometry and found a significant increase in the levels of H3K9ac (Fig 3D).

Lastly, we compared double siRNA transfection (5 days) with single siRNA transfection (3 days) and assessed the relative intensity of H3K9me3 using flow cytometry. Our results revealed that decreasing the time of siRNA transfection did not affect the relative intensity of H3K9me3 in the transfected cells. Single siRNA transfection was found to be adequately efficient to reduce the corresponding epigenetic mark in transfected cells (Fig 3E).

### The reconstruction of oocytes by siSUV39H1/H2 transfected cells results in a higher developmental potential than chaetocin-treated cells

Using siRNA (SUV39H1/H2)-transfected BFF cells as the somatic donor cells, we generated SCNT embryos and examined their pre-implantation development compared to SCNT embryos generated using chaetocin-treated BFFs. As shown in Table 2, despite there being no differences in terms of the cleavage rate between the siSUV39H1/H2, chaetocin and control groups, the siSUV39H1/H2 group demonstrated a significantly higher blastocyst yield (20.34±2.62) than the control (10.58±1.48) and chaetocin (10.13±1.29) groups (*P* < 0.05). This result confirms that H3K9 trimethylation acts as an epigenetic barrier in the nuclear reprogramming of bovine species, similar to mice and humans. In addition, to explore a relationship between histone hyperacetylation and reduced level of H3K9me3, we performed a combinatorial treatment of fibroblast cells with TSA and siSUV39H1/H2 and used them as donor cells in SCNT procedure (Table 3). Similar to our previous experiment (Table 1), TSA alone improved the blastocyst yield from 7.88±0.52% to 15.96±0.54% (approximately two fold increase). Combinational treatment of donor cells with TSA and siSUV39H1/H2 further increased the blastocyst rate to 28.5±3.07%, which is statistically higher than the TSA (15.96±0.54) and siSUV39H1/H2 (15.32±0.50) treatment alone (Table 3). Our result, unlike that of Matoba et al. [26] shows that TSA and siSUV39H1/H2 treatment have synergistic effect on blastocyst rate.

**Table 2:** Blastocyst development rate of the SCNT embryos derived from the siRNA SUV39H1/H2 transfected BFF cells.

Groups	No. of oocytes	Embryo development	
		% of Cleaved embryos ± SEM	% of blastocysts on day7 ± SEM
SCNT- Control	346	89.3±2.65 a	10.58±1.48 b
SCNT- siSUV39H1/H2	390	83.8±1.43 a	20.34±2.62 b
SCNT- Chaetocin 20nM	372	84.57±2.12 a	10.13±1.29 a

Values are presented as mean ± standard error of mean. Different letters indicate significant differences between the groups at *P*< 0.05.

**Table 3:** Blastocyst development rate of SCNT embryos derived from siRNA SUV39H1/H2 transfected cells, TSA treated cells and combination of siRNA SUV39H1/H2 and TSA.

Groups	No. of oocytes	Embryo development	
		% of Cleaved embryos ± SEM	% of blastocysts on day7 ± SEM
SCNT- Control	260	88.24±2.79 a	7.88±0.52 a
SCNT- siSUV39H1/H2	282	85.99±3.23 a	15.32±0.50 b
SCNT TSA (1 μM)	246	86.51±3.67 a	15.96±0.54 b
SCNT- siSUV39H1/H2 + TSA	291	90.25±3.50 a	28.85±3.07 c

Values are presented as mean ± standard error of mean. Different letters indicate significant differences between the groups at *P*< 0.05.

### siSUV39H1/H2 modifies the expression profile of genes of interest in the derived blastocysts similar to those derived from IVF

To further investigate the molecular mechanism through which siSUV39H1/H2 improves the developmental competence of the derived blastocysts, the transcription levels of three pluripotency genes (*POU5F1*, *NANOG*, and *SOX2*), two histone methyltransferase genes (*SUV39H1* and *SUV39H2*), and two DNA methyltransferase genes (*DNMT3A* and *DNMT3B*) were determined using quantitative PCR at the blastocyst stage.

**Fig 4:**
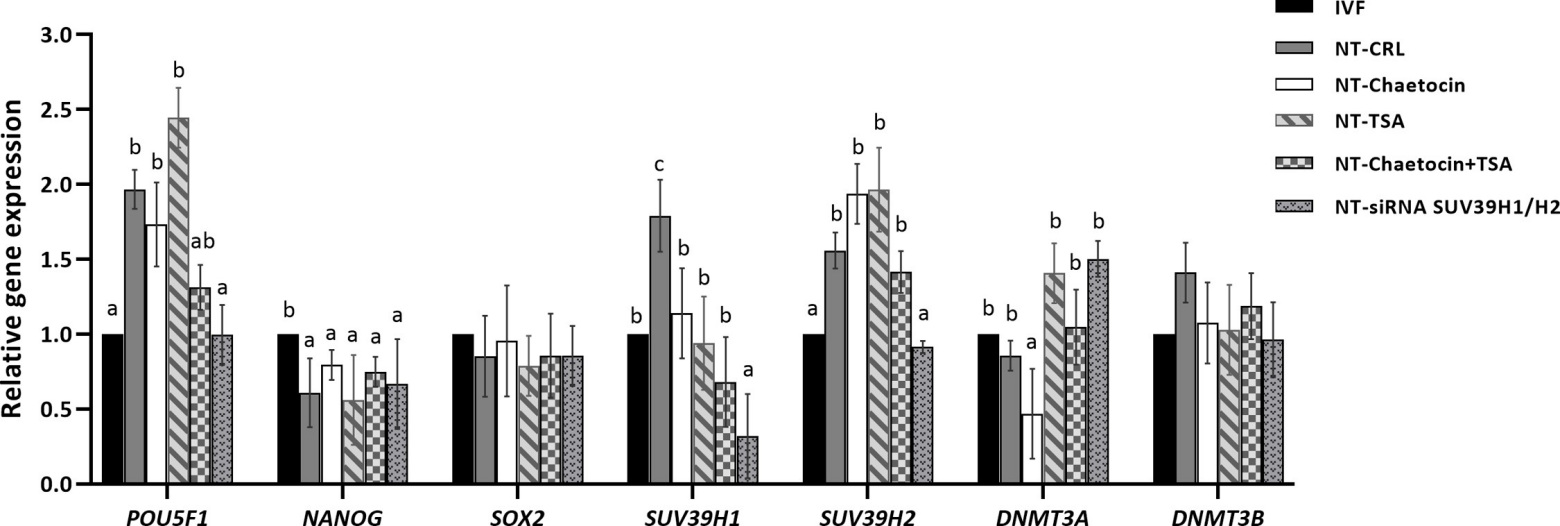
The relative gene expression of the developmentally important related genes. Real-time reverse-transcriptase PCR gene expression for the three pluripotency genes (*POU5F1*, *NANOG*, and *SOX2*), *SUV39H1/H2*, and *DNMT3A/B* in the blastocysts derived from the IVF, non-treated SCNT (SCNT-CRL) and chaetocin, TSA, chaetocin + TSA, and siSUV39H1/H2 treated SCNT. Fold-change values were calculated from three biological replications following normalization to *β-ACTIN*. Each biological replication contains six blastocyst in each group. Values are presented as mean ± standard error of mean. Different letters indicate significant differences between the groups at *P*< 0.05.

As shown in Fig 4, the relative expression level of *POU5F1* was reduced in the SCNT-siSUV39H1/H2 group compared to the other groups (*P* < 0.05), except for the IVF group, which showed a similar result (SCNT-siSUV39H1/H2 vs. IVF) (*P* > 0.05). The transcription level of *NANOG* was significantly lower in all the SCNT groups compared to the IVF group; siSUV39H1/H2 did not alter the expression of this gene (*P* > 0.05). The transcription of the last final pluripotency related gene, *SOX2*, was similar among all the experimental groups (*P* > 0.05).

We also assessed the expression of two important histone methyltransferases, namely SUV39H1 and SUV39H2, which mediate H3K9 trimethylation. While the expression of *SUV39H1* was significantly higher in the SCNT-control group compared to the IVF group (*P <* 0.05), the expression levels of this gene in the SCNT-chaetocin, SCNT-TSA, and SCNT-chaetocin + TSA groups were similar to the IVF group (*P* > 0.05). Furthermore, the expression of SUV39H1 in the SCNT-siSUV39H1/H2 group was significantly lower than that in the IVF group (*P* < 0.05). For *SUV39H2*, the transcription level in the SCNT-control, SCNT-chaetocin, SCNT-TSA, and SCNT-chaetocin + TSA groups was significantly higher than in the IVF group (*P* < 0.05), while the expression of this gene in the SCNT-siSUV39H1/H2 group was similar to that of the IVF group (*P* > 0.05).

Finally, we analyzed the expression of *DNMT3A* and *DNMT3B*, and found these genes to be expressed at similar levels in all the experimental groups (*P* > 0.05). Overall, the most similar transcription profile was observed between the IVF and siSUV39H1/H2 groups, which indicated that the siSUV39H1/H2 transfection of BFF cells not only played an effective role in the modification of the transcription profile of the derived SCNT blastocysts but also in developmental competence.

## Discussion

Fifty-five years after the first successful somatic cell nuclear transfer in *Xenopus* [37], the efficiency of this technique has remained low, especially in mammalian species, due to the strong resistance of somatic donor cells to epigenetic reprogramming and restart EGA [5].

H3K9me3, a repressive histone modification mediated by heterochromatin formation, acts as a reprogramming barrier to the generation of iPSCs [38]. This hypothesis has been supported by both loss-of-function and gain-of-function approaches, demonstrating that a reduction in H3K9me3 promotes the conversion of somatic cells into iPSCs. In two studies on mice and humans, Zhao’s group used comparative transcriptome and epigenomic analysis to show that reprogramming-resistant regions (RRRs) were enriched for H3K9me3 in somatic donor cells [27, 28]. They also reported that the removal of this epigenetic mark, either through the overexpression of *Kdm4d* (a specific demethylase of H3K9me3) in oocytes or by knocking-down *Suv39h1/2* (H3K9 methyltransferase) in donor cells, could improve the efficiency of SCNT with regards to blastocyst formation and live offspring yield.

According to these studies, Suv39h1/2-mediated H3K9me3 acts as an epigenetic barrier during epigenetic reprogramming in mammalian SCNT [27, 28] and represents a promising step for the improvement of SCNT efficiency compared to IVF. Based on these findings, the present study aimed to induce H3K9me3 removal in bovine fibroblast cells using chaetocin, a chemical inhibitor of Suv39h1/2.

Cell treatment with chaetocin for three days at non-toxic concentrations (10 or 20 nM) profoundly reduced H3K9me3 while increasing H3K9ac. In addition, in another experiment, we increased the time of chaetocin treatment from 3 days to 5 days. Our results, however, indicated that increasing the time of chaetocin treatment did not increase its effect on the relative intensity of H3K9me3 and H3K9ac. These results are consistent with those reported by Maleszewska et al. [39], who reported a similar decrease in H3K9me3 at all time-points in cells exposed to 10 nM chaetocin for 24, 48, and 72 h.

Consequently, we observed that the reduction in H3K9me3 after chaetocin treatment for 3 days did not improve the blastocyst yield (11.41±2.14% and 9.31±1.78%, respectively) compared to the control (9.62±1.25%).

To elucidate a potential relationship between the inhibition of histone deacetylase (HDAC) and SUV39H1/H2, fibroblast cells were treated with a combination of TSA and chaetocin. Similar to our previous reports [17, 18], TSA alone improved the blastocyst rate (17.5±0.98%), while a combination of TSA with chaetocin (16.1±1.18%) did not produce any additive effect. These results indicate that improvements in the blastocyst yield are solely due to TSA, such that treatment with chaetocin alone, or in combination with TSA, did not have any effect on SCNT reprogramming, at least during the blastocyst stage.

There are two possible explanations for these results: (1) Greiner and colleagues showed that chaetocin could efficiently inhibit *SUV39H1* [32], but other reports have indicated that chaetocin can also inhibit G9a and several other DNA/histone methyltransferases in a non-specific manner through chemical modification [36]. Insignificant improvements in blastocyst yield following the treatment of donor cells with chaetocin may be related to the non-specific inhibition of other enzymes by chaetocin. However, this should be assessed by evaluating the methylation pattern of other lyse residues in histones; (2) The marked improvement in blastocyst yield after the removal of H3K9me3 epigenetic marks in mice and humans raises the question as to whether the role of H3K9me3 as an epigenetic barrier is not conserved across all species.

To answer the latter question, we aimed to remove H3K9me3 by treating BFFs with *SUV39H1/H2* siRNA. We found that the treatment of fibroblasts with this siRNA improved the yield of blastocyst formation by 2-fold, confirming that somatic H3K9me3 (removal of H3K9me3 through siSUV39H1/H2) is an epigenetic barrier for SCNT-mediated reprogramming in bovine species. Notably, the observed differences in the results between the two treatments (chaetocin treatment for three days vs. double siSUV39H1/H2 transfection for five days) may have been due to differences in the length of the treatments.

Previous studies have demonstrated that H3K9me3 chromatin-rich regions bind to heterochromatin binding protein-1 (HP1) to recruit DNA methyltransferases (DNMTs), resulting in chromatin condensation that is resistant to reprogramming [40, 41]. In this regard, it has been shown that the downregulation of SUV39H1 histone methyltransferase removes histone deacetylases, such as SIRT1, from the chromatin bed, allowing the chromatin to relax and become more accessible to transcription factors [42–44]. These findings demonstrate the notion of a self-reinforcing repressive chromatin state acting through interactions with other epigenetic modifiers. With regard to this finding, in our study we observed a significant reduction in the expression of the family of *DNMTs* in the *SUV39H1/H2*-depleted fibroblast cells. The depletion of this repressive epigenetic marker and reduction in the mRNA expression of *DNMTs* may accelerate the positioning of activation marks, such as H3K9ac and H3K4me3, in the chromatin of the donor cells. Thus, the deposition of H3K9me3 initiates a more relaxed chromatin assembly that may lead to an increased accessibility for the reprogramming and transcriptional factors into the donor cells.

In contrast, we observed a non-significant increase in the mRNA expression levels of *SUV39H1/H2*, *DNMT1*, and *DNMT3B* genes, but not *DNMT3A*. This could be due to the fact that chaetocin inhibits SUV39H1/H2 activity but does not affect the mRNA expression of the DNA methyltransferases. Furthermore, the insignificant increase in relative mRNA abundance of DNMT family genes in chaetocin treated cells could be related to the non-specific action of chaetocin and may be a consequence of other affected epigenetic marks which can alter the mRNA expression of DNMTs family. These results, however, are in contradiction with the relationship between H3K9me3, HP1, and DNA methyltransferase family, and will require further investigations.

Meanwhile, our findings are supported by a previous study in which H3K9me3 was found to decelerate iPS cell generation in mice embryonic fibroblast (MEF) cells [26], in addition to its inhibitory effects during SCNT-mediated reprogramming in mice and humans [27, 28].

To further verify the beneficial effects of the downregulation of *SUV39H1/H2*, we compared the relative expression of several developmentally important genes between the IVF and siSUV39H1/H2 groups. Our gene expression analysis results illustrated that the relative expression of key genes in blastocysts from the siSUV39H1/H2 group was similar to that in IVF-derived blastocysts, indicating the beneficial effects of the removal of H3K9me3 on the gene expression pattern of derived blastocysts.

## Conclusion

In summary, the downregulation of *SUV39H1/H2* through siRNA in bovine fibroblast cells improved the yield and gene expression of the resulting blastocysts. However, this effect was not observed after the treatment of fibroblasts with chaetocin, a chemical inhibitor of SUV39H1/H2. The differences in the results between the two treatments (chaetocin treatment for three days vs. double siSUV39H1/H2 transfection for five days) may be due to the difference in the length of the treatments. It is possible that a reduction in repressive H3K9me3 marks resulted in a higher chromatin accessibility with greater reprogrammability. This improved efficiency correlated with a similar pattern of gene expression between the IVF and siSUV39H1/H2 groups. Our findings may provide a promising approach to improve the efficiency of mammalian cloning for agricultural and biomedical purposes.

## Supporting information

S1 Table (DOCX)

S2 Table (DOCX)

S3 Table (DOCX)
