# Supplementary material for: siRNA inhibition and not chemical inhibition of Suv39h1/2 enhances pre-implantation embryonic development of bovine somatic cell nuclear transfer embryos
Source: PLoS One. 2020 Jun 4;15(6):e0233880. doi: 10.1371/journal.pone.0233880 (PMC7272017; doi:10.1371/journal.pone.0233880)
Supplement: S1 Table — (DOCX) [file pone.0233880.s001.docx]

| Name | Sequences (5'-3') |
| --- | --- |
| siSUV39H1 | Sense: GCCCAUACCCAAAGUAGAA- dTdT  Antisense: UUCUACUUUGGGUAUGGGC- dTdT |
| siSUV39H2 | Sense: GUGCAACUCAAGAUGUCAA- dTdT  Antisense: UUGACAUCUUGAGUUGCAC- dTdT |
| siSCR | Sense: GCAGUUACACCUCGGCUAU- dTdT  Antisense: AUAGCCGAGGUGUAACUGC- dTdT |

**Table 1. Bovine SUV39H1 and SUV39H2 siRNA sequences.**
