# Supplementary material for: siRNA inhibition and not chemical inhibition of Suv39h1/2 enhances pre-implantation embryonic development of bovine somatic cell nuclear transfer embryos
Source: PLoS One. 2020 Jun 4;15(6):e0233880. doi: 10.1371/journal.pone.0233880 (PMC7272017; doi:10.1371/journal.pone.0233880)
Supplement: S2 Table — (DOCX) [file pone.0233880.s002.docx]

| Antibody | Supplier | Catalog number | Species | Type | Dilution |
| --- | --- | --- | --- | --- | --- |
| Anti-acetyl-Histone H3 (Ac-Lys9) antibody (Primary antibody) | Sigma | H0913 | Mouse | Monoclonal/IgG | 1/200 |
| Anti-Histone H3 (tri methyl K9) antibody (Primary antibody) | Abcam | Ab8898 | Rabbit | Monoclonal/IgG | 1/100 |
| Goat Anti-Mouse IgG antibody, (H+L) FITC conjugated (Secondary antibody) | Chemicon | AP124F | Goat | Polyclonal/IgG | 1/50 |
| Goat Anti-Rabbit IgG (whole molecule), F(ab′)2 fragment–FITC Conjugated (Secondary antibody) | Sigma | F1262 | Goat | Polyclonal/IgG | 1/80 |

Table 2: List of antibodies used for immunofluorescence flowcytometry.
