# Supplementary material for: siRNA inhibition and not chemical inhibition of Suv39h1/2 enhances pre-implantation embryonic development of bovine somatic cell nuclear transfer embryos
Source: PLoS One. 2020 Jun 4;15(6):e0233880. doi: 10.1371/journal.pone.0233880 (PMC7272017; doi:10.1371/journal.pone.0233880)
Supplement: S3 Table — (DOCX) [file pone.0233880.s003.docx]

| Gene | Forward primer (5'- 3') | Reverse primer (5'- 3') | AT (°C) | Product size | Accession number |
| --- | --- | --- | --- | --- | --- |
| *B-ACTIN* | TTCCTGGGTATGGATCCTG | GGTGATCTCCTTCTGCATCC | 58 | 130 | [XM_015467124.1](https://www.ncbi.nlm.nih.gov/entrez/viewer.fcgi?db=nucleotide&id=982998074) |
| *DNMT1* | GAAGCAGAATAAGAATCGG | TTTGAAGAGTCGTCTGGAA | 54 | 144 | [NM_182651.2](https://www.ncbi.nlm.nih.gov/nuccore/NM_182651.2) |
| *DNMT3A* | TGGTCCTGGGCGTTAG | CCTGCTTTATGGAGTTCG | 57 | 252 | [NM_001206502.1](https://www.ncbi.nlm.nih.gov/entrez/viewer.fcgi?db=nucleotide&id=330417959) |
| *DNMT3B* | CGTCATCGCCCAGTGT | TCTTCTCCCTCGCCATCT | 54 | 195 | [NM_181813.2](https://www.ncbi.nlm.nih.gov/nuccore/NM_181813.2) |
| *GAPDH* | GTTCAACGGCACAGTCAAG | TACTCAGCACCAGCATCAC | 60 | 115 | - |
| *NANOG* | TTGTGACGGCTATTGTATG | ACCTCTTACTGGACTCATT | 53 | 159 | [NM_001025344.1](https://www.ncbi.nlm.nih.gov/entrez/viewer.fcgi?db=nucleotide&id=70778751) |
| *POU5F1* | GGAAAGGTGTTCAGCCA | ATTCTCGTTGTTGTCAGC | 62 | 123 | [NM_174580.3](https://www.ncbi.nlm.nih.gov/entrez/viewer.fcgi?db=nucleotide&id=1018191598) |
| *SOX2* | ATGGGCTCGGTGGTG | CTCTGGTAGTGCTGGGA | 57 | 182 | [NM_001105463.2](https://www.ncbi.nlm.nih.gov/nuccore/NM_001105463.2) |
| *SUV39H1* | GCGAACAGGAGTATTACC | TGGAACTGCTTGAGAATG | 55 | 106 | [NM_001046264.2](https://www.ncbi.nlm.nih.gov/nuccore/NM_001046264.2) |
| *SUV39H2* | ACCTGCTGTTGCTGAATAC | GTGAGTCTTCCTTCTGTTGAG | 61 | 91 | [NM_001037479.2](https://www.ncbi.nlm.nih.gov/nuccore/NM_001037479.2) |

Table 3: List of primers used in this study for real time PCR
